# Comparative Effects of Quercetin and its α- and β-D-glucoside Derivatives in LPS-Stimulated C6 Astroglial Cells

**DOI:** 10.1007/s11064-026-04875-8

**Published:** 2026-08-31

**Authors:** Michele Goulart dos Santos, Tainá Guillante, Rafael Felipe de Aguiar, Marcelo Augusto Germani Marinho, Diele Bopsin da Luz, Marie Demonceaux, Claude Solleux, Lucia Emanueli Schimith, Carlos Eduardo da Rosa, Corinne André-Miral, Bruno Dutra Arbo, Mariana Appel Hort

**Affiliations:** 1 Programa de Pós-Graduação em Ciências Fisiológicas, Instituto de Ciências Biológicas, Universidade Federal do Rio Grande - FURG, Campus Carreiros, Rio Grande, RS 96201900 Brazil; 2 Unit at the Biological Sciences at Biotechnologies, Nantes University, Nantes, France; 3 Programa de Pós-Graduação em Ciências da Saúde, Faculdade de Medicina, Universidade Federal do Rio Grande, Rio Grande, Rio Grande do Sul Brazil; 4 Departamento de Farmacologia, Instituto de Ciências Básicas da Saúde, Universidade Federal do Rio Grande do Sul, Porto Alegre, Rio Grande do Sul Brazil

**Keywords:** Neuroinflammation, Glucosylation, Flavonoid derivatives, Anti-inflammatory activity

## Abstract

Neuroinflammation is a response of the central nervous system to injury, infection, or disease, involving the activation of glial cells and the release of cytokines and reactive species. When sustained or dysregulated, it contributes to the progression of neurological disorders. Quercetin (Que) is one of the most extensively studied flavonoids; however, its therapeutic application is limited by low bioavailability. Glucosylation has therefore emerged as a strategy to improve the physicochemical and biological properties of flavonoids. Previously, we synthesized the derivative quercetin-3’-α-D-glucoside (Que-3α) and characterized its pharmacokinetic profile and in vitro cytotoxicity. Here, we investigated the anti-inflammatory and antioxidant effects of Que and its derivatives, including the recently synthesized Que-3α and the naturally occurring β-glucoside, quercetin-3-β-D-glucoside (Que-3β), in a lipopolysaccharide (LPS)-induced inflammation model using C6 astroglial cells. Cells were pretreated for 2 h with the compounds (15 and 30 µM) and then stimulated with LPS (10 µg/mL) for 24 h. The compounds preserved mitochondrial activity, while Que and Que-3α reduced LPS-induced reactive oxygen species production, increased cell viability, and decreased apoptosis and necrosis. Notably, the Que-3α reduced iNOS expression, indicating more effective modulation of inflammatory mediators compared with the aglycone. These findings reinforce the biological properties of Que and suggest that α-glucosylation preserves the beneficial actions of Que while enhancing pharmacodynamic features relevant to neuroinflammation.

**Graphical Abstract:**

**Figure:**
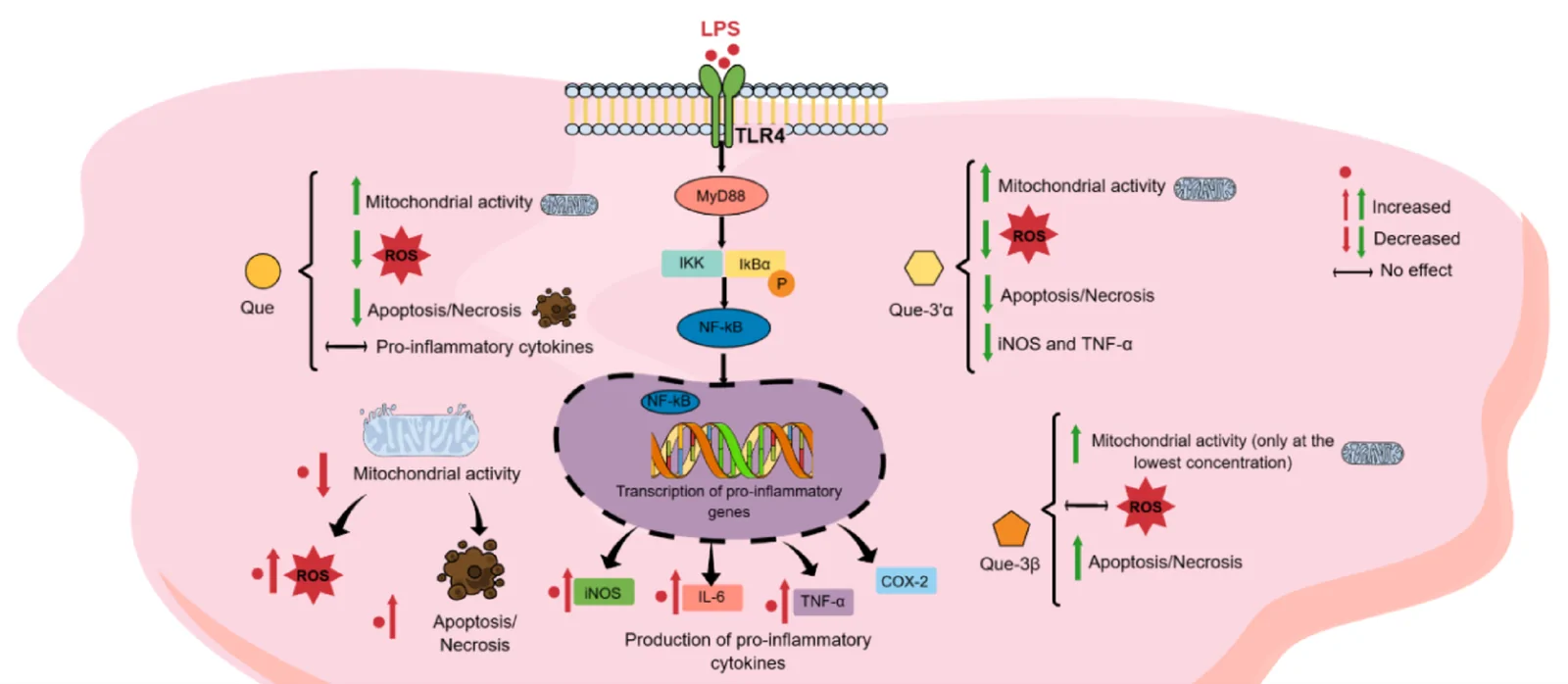

## Introduction

Neuroinflammation is a process that involves the coordinated activation of innate and adaptive immune responses within the central nervous system (CNS), triggered by various harmful insults such as infection, ischemia, stress, and trauma [1]. This process is characterized by the release of inflammatory mediators, including cytokines, chemokines, and reactive oxygen species (ROS), by different cell types, mainly microglia and astrocytes [2]. Research indicate that a robust inflammatory response in the peripheral system, triggered by factors such as systemic exposure to lipopolysaccharide (LPS) or viral infections, can result in the infiltration of immune cells from the periphery into the CNS [3, 4]. This infiltration then leads to neuroinflammation and nerve cell destruction due to increased permeability of the blood-brain barrier (BBB) [5].

Although this process plays a protective and reparative role in its early stages, evidence from clinical and preclinical studies indicate that its prolonged or excessive activation significantly contributes to the pathogenesis of several neurological disorders, constituting a common link among different conditions of the CNS, such as ischemic, degenerative, traumatic, demyelinating, epileptic, and psychiatric pathologies [6]. These diseases are highly prevalent and constitute major public health concerns, given their association with high morbidity, significant negative impact on individuals quality of life, the complexity of clinical management, and elevated socioeconomic costs [7]. A study published in The Lancet Neurology [8] reported that over 3 billion people worldwide were affected by a neurological condition in 2021, establishing CNS pathologies as the leading cause of disease burden and disability globally [9].

Conventional chronic treatments for inflammatory conditions, whether peripheral or central, rely mainly on non-steroidal anti-inflammatory drugs (NSAIDs) and corticosteroids [10]. However, their long-term use is limited by well-documented adverse effects. NSAIDs can lead to gastric and duodenal ulceration and, in severe cases, gastrointestinal bleeding [11]. High doses of glucocorticoids are associated with bone loss and osteoporosis [12], as well as muscle catabolism through protein degradation and inhibition of protein synthesis [13]. Additional systemic consequences include metabolic disturbances, such as hypertension and obesity [14], and behavioral or cognitive impairments, including memory deficits [15]. These risks justify the increasing interest in investigating natural compounds with anti-inflammatory properties and improved pharmacological safety.

**Fig. 1:**
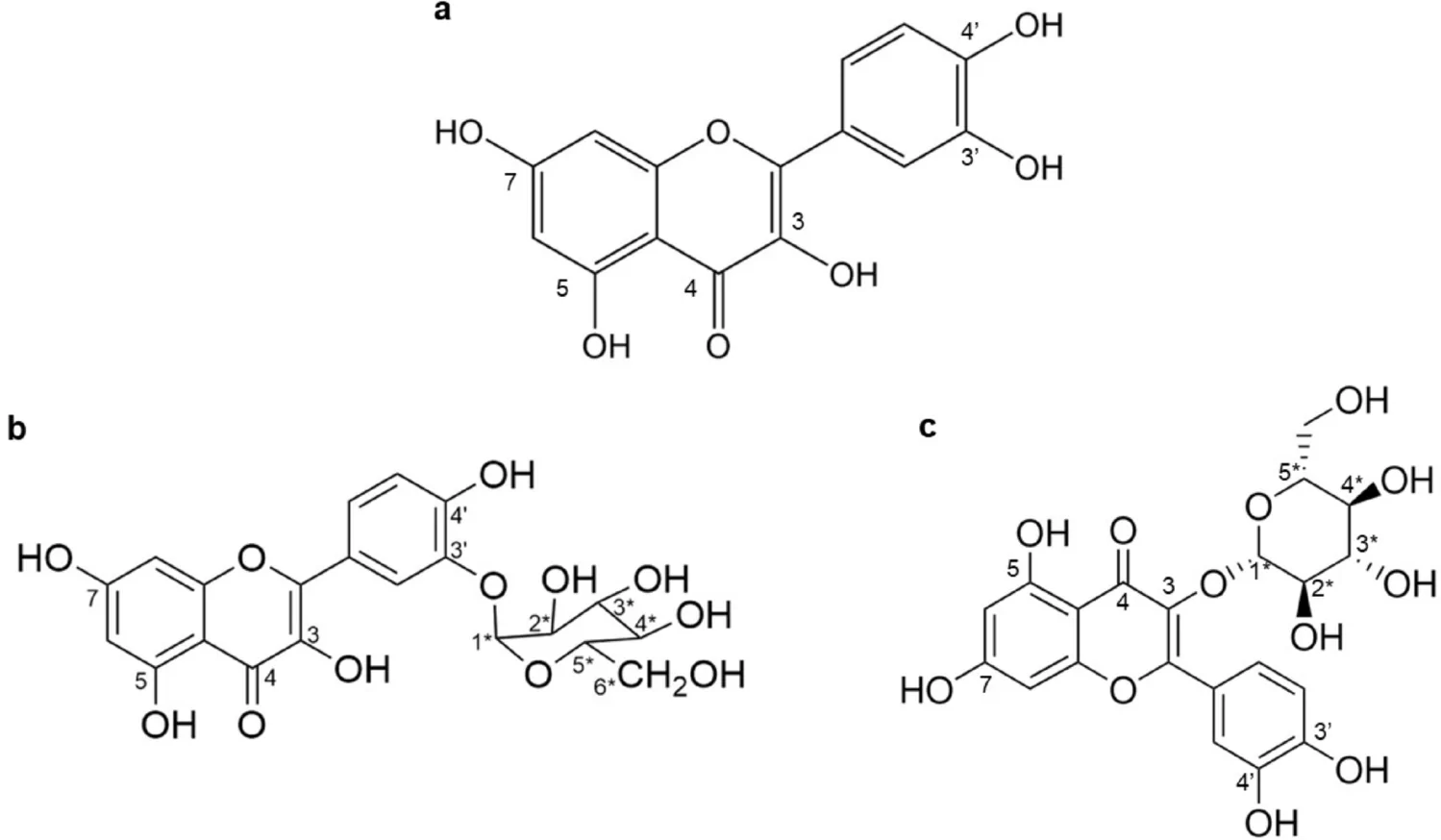
Chemical structure of quercetin (**a**) quercetin-3’-α-D-glucoside derivative (**b**), and quercetin 3-β-D-glucoside (**c**)

Flavonoids represent a class of molecules with diverse neuroprotective activities, exerting both antioxidant and anti-inflammatory effects. Among them, quercetin (Que) (3,3′,4′,5,7-pentahydroxyflavone) (Fig. 1A) stands out as a dietary flavonoid formed by three aromatic rings and five hydroxyl groups. Abundant in fruits and vegetables, this compound has been extensively recognized for its antioxidant [16], antiviral [17], anticancer [18], and anti-inflammatory [19, 20] properties.

Previous studies have reported that Que can reduce the production of pro-inflammatory cytokines, such as tumor necrosis factor alpha (TNF-α) and some interleukins, including IL-1β and IL-6 [21], inhibits nitric oxide (NO) release [22]. Experimental evidence has also suggested that these effects may involve modulation of signaling pathways associated with neuroinflammation, including NF-κB and MAPK [23, 24], attenuation astrocyte and microglial activation [25, 26] and enhances antioxidant defenses by upregulating enzymes such as superoxide dismutase (SOD) and catalase (CAT), thereby mitigating oxidative stress [27, 28].

Despite these beneficial effects, Que exhibits low water solubility and poor bioavailability, which limit its clinical application [29]. To address these limitations, recent studies have focused on developing advanced delivery systems, including nanoparticles and microemulsions [30]. Moreover, previous studies have reported that glucosylated and sulfated derivatives, may exhibit improved bioavailability while maintaining biological activities associated with anti-inflammatory and antioxidant effects [31–34].

Glucosylation has been proposed as a promising strategy to modulate the physicochemical properties of bioactive compounds [35]. This structural modification can influence the molecule’s ability to cross biological barriers, such as the BBB, making it a potential candidate for neuroprotective effects in the context of neuroinflammation [36]. Our research group has been dedicated to the synthesis and characterization of new glucosylated derivatives of different flavonoids [37–40]. Unlike the naturally occurring β-glycosides of Que, such as quercetin 3-O-β-D-glucoside (isoquercitrin) (Fig. 1C), the molecules synthesized in this work incorporate glycosidic linkages in the α-configuration. This structural modification is not commonly found in nature and may confer distinct physicochemical and biological properties compared to the aglycone. A recent example is the glucosylated derivative Quercetin-3’-α-D-glucoside (Que-3α), which contains a glucose unit attached to the 3-α carbon of the molecule (Fig. 1B). In a previous study, we characterized the pharmacokinetic properties of this derivative, as well as its in vitro cytotoxicity [41].

In the present study, we investigated the anti-inflammatory effects of Que and its novel glucosylated derivative, Que-3α, against LPS-induced damage in rat C6 astroglial cells. Additionally, the β-glucosylated derivative Que-3β (isoquercitrin) was evaluated for comparison. Our aim was to evaluate whether the structural modification, through sugar addition, alters the protective effects of the molecule compared to the aglycone.

## Materials and Methods

### Chemicals

Quercetin (CAS No. 117-39-5), quercetin 3-O-β-D-glucoside (isoquercitrin; cat. No. 17793), lipopolysaccharide (LPS from *Escherichia coli* O111:B4; cat. No. L3024), 3-(4,5-dimethylthiazol-2-yl)−2,5-diphenyltetrazolium bromide (MTT; CAS No. 298-93-1), acridine orange (cat. No. A9231), propidium iodide (cat. No. P4170), and 2′,7′-dichlorodihydrofluorescein diacetate (H₂DCFDA; cat. No. D6883) were obtained from Sigma-Aldrich (St. Louis, MO, USA). Dimethyl sulfoxide (DMSO; CAS No. 67–68-5), Dulbecco’s Modified Eagle Medium (DMEM; cat. No. 31600-034), Fetal Bovine Serum (FBS; cat. No. 12657-029), Trypsin-EDTA (0.5%) (10×; cat. No. 15400-054), and Antibiotic-Antimycotic solution (100×; cat. No. 15240-062) were obtained from Gibco (Thermo Fisher Scientific, Carlsbad, CA, USA). TRIzol^®^ reagent (Invitrogen – cat. No. 15596026), Chloroform (CAS No. 67-66-3), isopropyl alcohol (CAS No. 67-63-0), ethanol (CAS No. 64-17-5), and DNase/RNase-free water were used for RNA extraction by the organic method. All reagents were of analytical grade.

### Enzymatic Synthesis of the Quercetin-3’-α-D-glucoside

Que-3α was synthesized by enzymatic transglucosylation employing the modified sucrose phosphorylase BaSP Q345F, as previously described in dos Santos et al. [41]. Briefly, the enzyme was expressed in *Escherichia coli* BL21(DE3) carrying a pET28b vector encoding the BaSP variant and purified by Ni-NTA affinity chromatography, as previously reported [40]. The transglucosylation reactions were performed in MOPS–NaOH buffer (50 mM, pH 8.0) containing Que, sucrose as the glucose donor, and the purified BaSP Q345F enzyme, followed by incubation at 37 °C for 24 h. The reaction mixture was analyzed by analytical HPLC, and the resulting α-glucosylated Que derivative was subsequently isolated by preparative HPLC. Structural confirmation was obtained by high-resolution mass spectrometry and by ¹H and ¹³C NMR spectroscopy.

### In Vitro Assays

#### Cell Culture

The C6 rat astroglial cell line was obtained from the Rio de Janeiro Cell Bank. Cells were cultured in DMEM supplemented with 10% fetal bovine serum, 2 mM L-glutamine, 1 mM HEPES, 4.5 g/L glucose, and 10,000 IU/mL penicillin and 10,000 IU/mL streptomycin. Cultures were maintained in a humidified atmosphere at 37 °C with 5% CO₂, at 70–80% confluency and within 30 passages. The cell line was mycoplasma-free.

#### Experimental Design

**Fig. 2:**
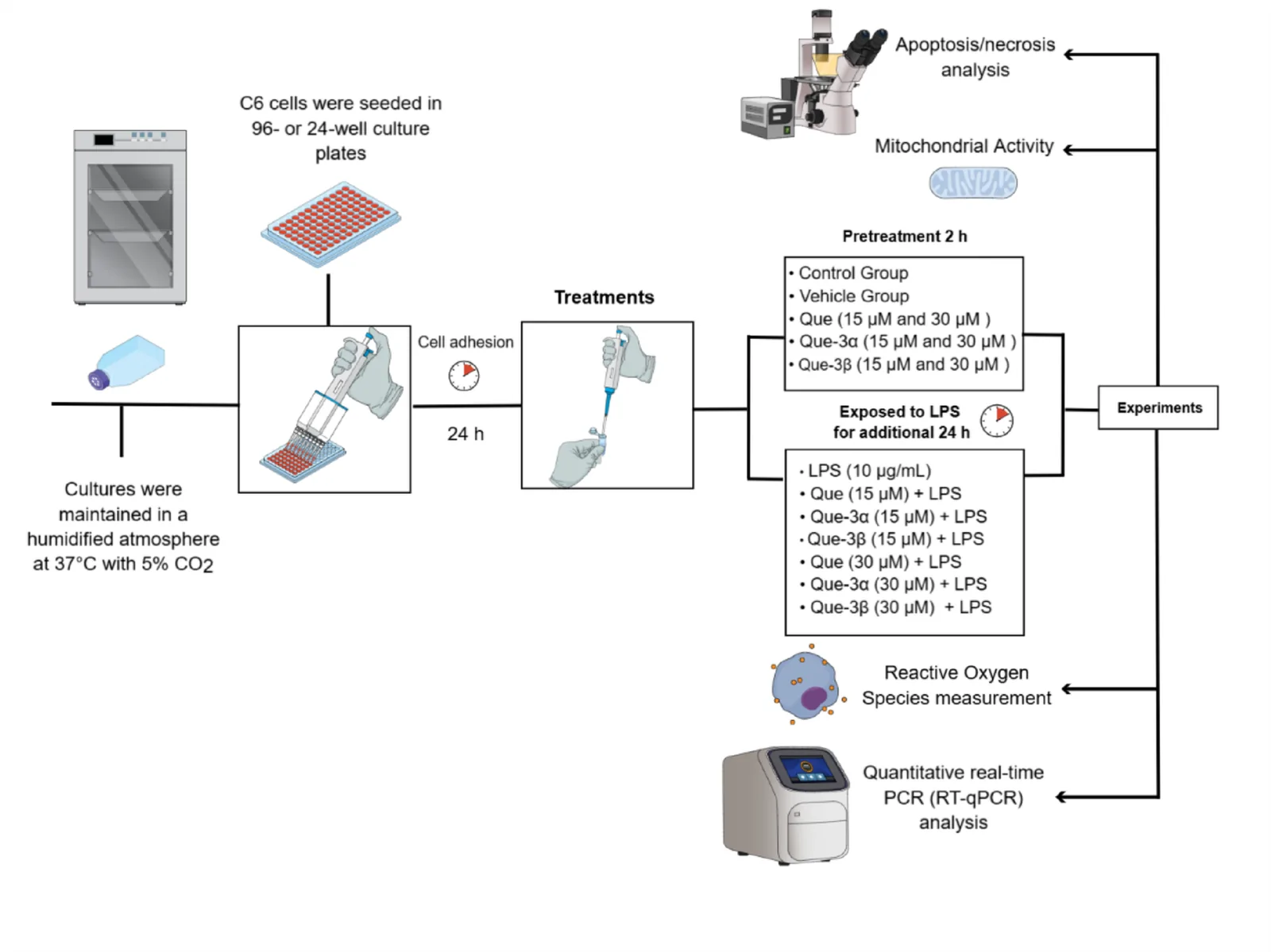
Schematic representation of the experimental design used to evaluate the effects of quercetin (Que), quercetin-3’-α-glucosylated (Que-3α) and quercetin 3-β-D-glucoside (Que-3β) on C6 astroglial cells. After seeding and 24 h of adhesion, the cells were pretreated for 2 h with Que, Que-3α or Que-3β (15 or 30 µM), followed by stimulation with LPS (10 µg/mL) for 24 h. Subsequent analyses included the evaluation of mitochondrial activity, apoptosis/necrosis, reactive oxygen species (ROS) production, and expression of inflammatory mediators by RT-qPCR

C6 cells were seeded in 96-well plates with a final volume of 100 µL per well or in 24-well plates with a final volume of 250 or 500 µL per well, at densities of 7 × 10³ cells/well or 7 × 10⁴ cells/well, respectively, according to the requirements of each assay. Following seeding, cultures were incubated for 24 h to allow cell adhesion before the treatments. After this period, cells were pre-treated for 2 h with Que, Que-3α or Que-3β (15 or 30 µM). Stock solutions of Que (50 mM), Que-3α and Que-3β (15 mM) were prepared in DMSO (100%), and working solutions were prepared in serum-free culture medium. The concentrations were selected based on a previous study of cytotoxicity in the same cell line [41]. Subsequently, cells were exposed to LPS (10 µg/mL) for additional 24 h. The experimental groups included: control (DMEM), vehicle (0.06% DMSO), Que 15 µM, Que 30 µM, Que-3α 15 µM, Que-3α 30 µM, Que-3β 15 µM, Que-3β 30 µM, LPS (10 µg/mL), Que 15 µM + LPS, Que 30 µM + LPS, Que-3α 15 µM + LPS, Que-3α 30 µM + LPS, Que-3β 15 µM + LPS and Que-3β 30 µM + LPS. At the end of the exposure period, different analyses were performed. Cells cultured in 96-well plates were used for mitochondrial activity assays and apoptosis/necrosis evaluation, whereas cells grown in 24-well plates were collected for ROS quantification and gene expression analysis by RT-qPCR. All experiments were performed in triplicate, except for the PCR analyses, which were performed in 6 replicates. The experimental design is illustrated in Fig. 2.

#### Mitochondrial Activity Assay

Mitochondrial activity assay was assessed using the 3-[4,5-dimethylthiazol-2-yl]−2,5-diphenyltetrazolium bromide (MTT) assay as described by Mosmann [42], which evaluates mitochondrial metabolic activity. C6 cells were seeded and subsequently treated under the conditions described above, and the MTT assay was performed after the treatment period. Five µL of MTT solution (5 mg/mL) was added to each well and incubated for 2 h at 37 °C. The culture medium was then carefully removed, and the resulting insoluble purple formazan crystals were dissolved in 150 µL of DMSO. Absorbance was measured at 550 nm, using a microplate reader (ELx800 Microplate Reader, Biotek Instruments, Vermont, USA), Treatments were performed in triplicate in three independent experiments. Data on cell viability were expressed as a percentage of control group.

#### Reactive Oxygen Species (ROS) Measurement

For ROS analysis, as described by Myhre and Fonnum [43], the culture medium was removed, and the cells were detached using trypsin and transferred to individually labeled microtubes. The samples were then spun at 206 x g for 5 min, the supernatant was thrown away, and the cell pellet was washed once with PBS. Subsequently, the cells were resuspended in PBS containing the fluorescent probe 2′,7′-dichlorofluorescein diacetate (H₂DCF-DA; 40 µM) and incubated for 30 min at 37 °C in the dark. After incubation, the samples were centrifuged (206 x g, 5 min), washed twice with PBS, and resuspended in 1 mL of PBS. Aliquots of 160 µL of the cell suspension were transferred, in triplicate, to white-walled microplates for fluorescence measurement. Blanks containing only PBS and H₂DCF-DA were also prepared in triplicate. Fluorescence intensity was measured using a microplate reader (Victor², PerkinElmer, USA) at excitation/emission wavelengths of 485/520 nm for 90 min at 37 °C. The data were normalized to the number of viable cells at the end of the analysis, which were quantified using the 0.4% trypan blue exclusion method (1:1, v/v) and counted in a Neubauer chamber blindly by an experimenter. Results were then expressed as fluorescence units per minute (FU·min⁻¹/viable cells).

#### Apoptosis/Necrosis Analysis

Cell death and viability analysis was performed using the Acridine Orange (AO) and Propidium Iodide (PI) staining method, based on modifications of the protocol described by Ng et al. [44]. AO gives off green fluorescence and can penetrate into all cells, staining the DNA of cells that are still alive. In contrast, PI emits red fluorescence and cannot pass through intact membranes. This means that it only marks cells with damaged membranes, which means that the cells are dead from necrosis or late apoptosis [45].

C6 cells were seeded and treated under the same conditions mentioned previously, but for this analysis only the highest concentrations of the molecules were used. After treatments, 10 µL of AO (100 µg/mL) and PI 100 µg/mL (1:1, v/v) were added to each well and left to sit for 2 min. Cells were washed with PBS to eliminate residual dye, and the cells were immediately observed using an inverted epifluorescence microscope (Olympus IX81- Olympus Corporation, Tokyo, Japan) at 200x magnification. Three random fields per well were photographed, and subsequently, the images were quantified using ImageJ software (version 1.53, National Institutes of Health, USA) by an investigator blinded to the experimental groups. Cells were classified into three distinct stages based on their fluorescence emission and morphological appearance: (1) Viable Cells (uniform bright green nucleus with organized cellular structures); (2) Apoptotic Cells (nucleus presenting a yellowish or orange/red color, with signs of chromatin condensation and/or fragmentation and membrane blebbing); and (3) Necrotic/late apoptosis Cells (uniformly red nucleus, with preserved structures and absence of chromatin condensation). The assay was performed in triplicate, and data were expressed as the percentage of viable, apoptotic, and necrotic cells relative to the total number of cells counted (total sum of viable, apoptotic, and necrotic cells).

#### RNA Isolation and cDNA Synthesis

Total RNA was extracted from the cells using TRIzol^®^ reagent (Invitrogen) according to the manufacturer’s instructions. The contents of four wells, each containing 250 µL of the cell suspension with TRIzol^®^, were combined into a single microtube, resulting in a final volume of 1 mL per sample. Only the 30 µM concentrations of Que and Que-3α were used for the RT-qPCR analyses. RNA integrity was assessed by standard agarose gel electrophoresis. RNA quantification was performed using a BioDrop (BioDrop Ltd., Cambridge, UK), and RNA purity was determined by the absorbance ratio (AR) at 260 and 280 nm. Only high-purity samples (AR 260/280 ≥ 1.8) were used for subsequent steps. For cDNA synthesis, all total RNA concentrations were normalized to the lowest concentration value, and the GoScript™ Reverse Transcription System (Promega, USA) was utilized following the manufacturer’s recommendations.

#### Quantitative Real-Time PCR (RT-qPCR) Analysis

RT-qPCR reactions were performed to quantify gene expression using the GoTaq^®^ qPCR Master Mix (Promega, USA). All reactions were carried out in duplicate, with a final volume of 10 µL per well, containing 0.5 µL of forward primer, 0.5 µL of reverse primer, 2 µL of cDNA, and 8 µL of the master mix.

Gene-specific primers for the transcripts expressed in C6 cells (Table 1) were designed using the Primer-BLAST tool, based on gene sequences available in the National Center for Biotechnology Information database (NCBI; https://www.ncbi.nlm.nih.gov/). The amplification protocol consisted of an initial denaturation step at 95 °C for 2 min, followed by 40 cycles of denaturation at 95 °C for 15 s and annealing/extension at 60 °C for 1 min.

Two reference genes (β-actin and Tubulin-1) were used for normalization. Primer efficiency was determined from standard curves generated using five serial dilutions of cDNA, and only primers with efficiency between 90% and 110% were considered acceptable. Relative gene expression levels were calculated using the E⁻^ΔΔCt^ method, as described by Schmittgen and Livak [46].

**Table 1:** Primer sequences used to evaluate gene expression by real-time PCR in rat astroglial cells (*Rattus norvegicus*)

Nº	Gene	Protein	Primers (5’−3’)	GenBank
1	*actb1*	Beta actin 1	F: CGCGAGTACAACCTTCTTGC R: CGTCATCCATGGCGAACTGG	NM_031144.3
2	*Tuba1a*	Tubulin, α 1 A	F: CGCTGTAAGAAGCAACACCT R: GGAGATACACTCACGCATGG	NM_022298.1
3	*TNF-α*	Tumor necrosis factor α	F: GATCGGTCCCAACAAGGAGG R: CTTGGTGGTTTGCTACGACG	NM_012675.3
4	*Il-6*	interleukin 6	F: GCCCACCAGGAACGAAAGTC R: TGGCTGGAAGTCTCTTGCGG	NM_012589.2
5	*iNOS*	Inducible nitric oxide synthase	F: AGAATCCCTGGACAAGCTGC R: CTTGTGGTGAAGGGTGTCGT	Mahmoud et al. [47]
6	*Ptgs2* *(COX-2)*	Prostaglandin-endoperoxide synthase 2	F: ACGTGTTGACGTCCAGATCA R: GGCCCTGGTGTAGTAGGAGA	NM_017232.4

### Statistical Analysis

The distribution of the data was assessed for normality using the Shapiro–Wilk and Kolmogorov–Smirnov tests. Differences among groups were evaluated using one-way analysis of variance (ANOVA) followed by *post hoc* of Tukey for multiple comparisons. Data are expressed as mean ± standard error of the mean (SEM), and differences were considered statistically significant at *p* < 0.05. All analyses were performed using GraphPad Prism software, version 9.0 (GraphPad Software, San Diego, CA, USA).

## Results

###  Quercetin and the Derivative Quercetin-3’-α-D-glucoside and Quercetin 3-β-D-glucoside Preserve Mitochondrial Activity

**Fig. 3:**
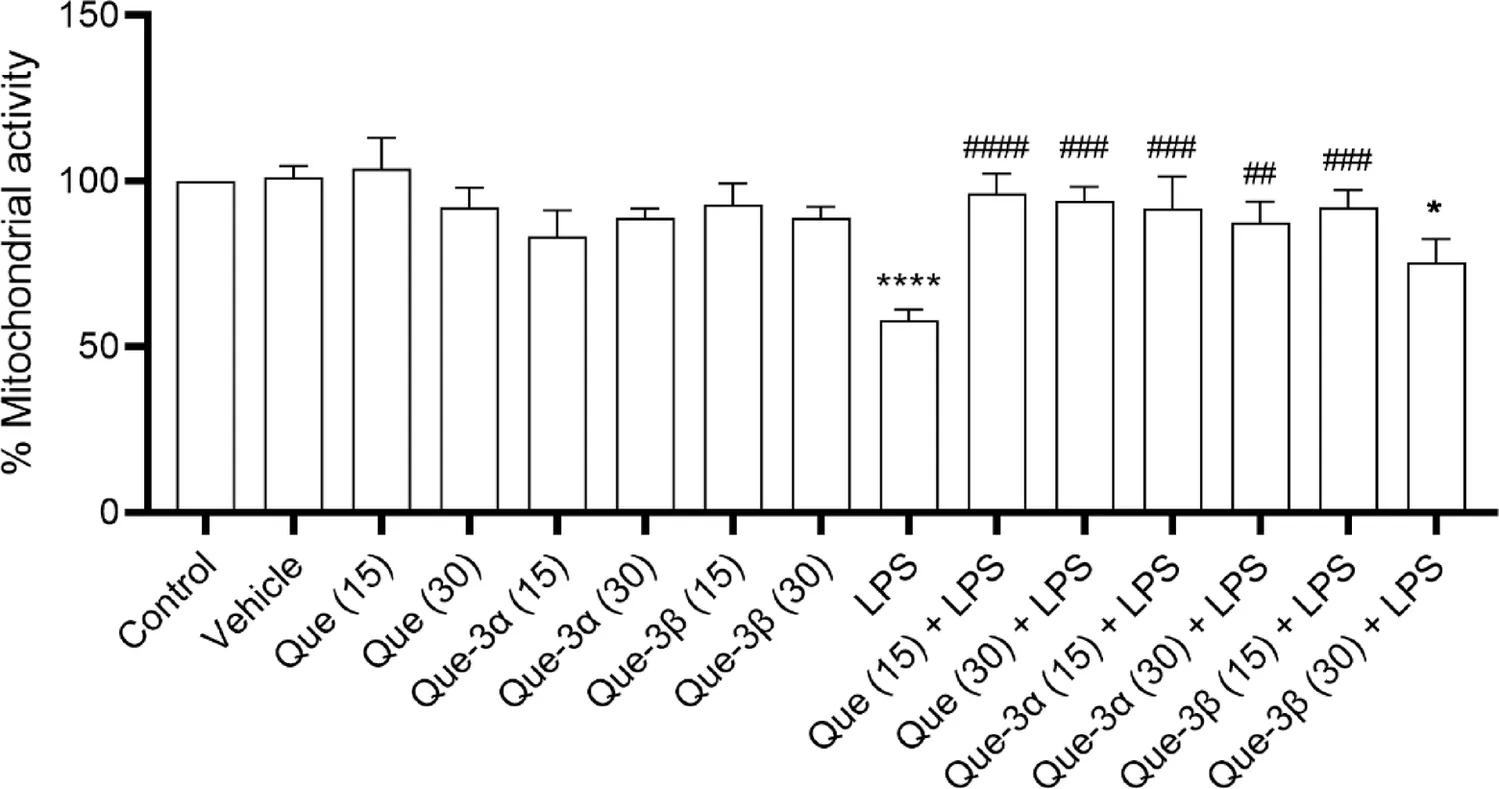
Effects of quercetin (Que), quercetin-3’-α-D-glucoside (Que-3α) and quercetin 3-β-D-glucoside (Que-3β) on mitochondrial activity in LPS-stimulated C6 cells. Cells were pre-treated with Que or Que-3α or Que-3β (15 or 30 µM) or vehicle (DMSO 0.06%) for 2 h and then exposed to LPS (10 µg/mL) for additional 24 h. Data are presented as mean ± SEM of at least three independent experiments. ****p < 0.001 vs. Vehicle; #*p* < 0.05, ##*p* < 0.01 and ###*p* < 0.001 vs. LPS. One-way ANOVA followed by Tukey post-test for multiple comparisons

Metabolically active cells were assessed by MTT assay (Fig. 3). Exposure to LPS significantly reduced mitochondrial activity compared to the control group, confirming its cytotoxic effects. Treatment with Que or Que-3α and Que-3β in the two tested concentrations did not alter mitochondrial activity when administered alone, indicating no cytotoxicity. The pre-treatment with Que and Que-3α 15 µM and 30 µM significantly attenuated the LPS-induced reduction in mitochondrial activity, suggesting a protective effect on mitochondrial function. There were no significant differences between the molecules. For Que-3β, only the lowest concentration (15 µM) restored mitochondrial activity in cells exposed to LPS, indicating a protective effect, while the highest concentration (30 µM) failed to prevent the LPS-induced reduction. No significant differences were observed between the molecules under equivalent conditions (Fig. 3).

### Quercetin and the Derivative Quercetin-3’-α-D-glucoside Prevent LPS-Induced ROS Production in C6 Cells

**Fig. 4:**
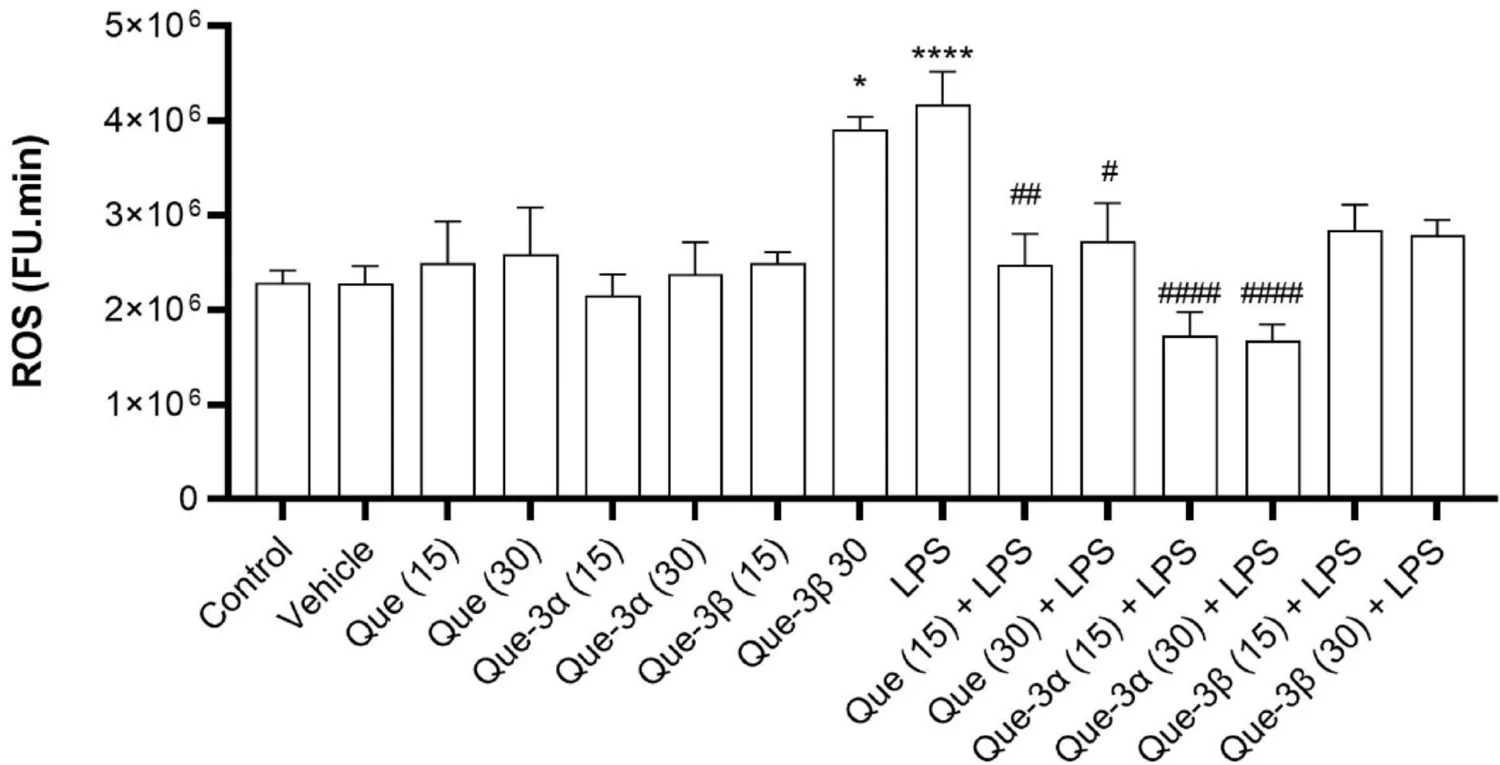
Effect of quercetin (Que), quercetin-3’-α-D-glucoside (Que-3α) and quercetin 3-β-D-glucoside (Que-3β) on reactive oxygen species (ROS) production in C6 cells. Cells were pre-treated with Que, Que-3α or Que-3β (15 or 30 µM) or vehicle (DMSO 0.06%) for 2 h and then exposed to LPS (10 µg/mL) for additional 24 h. ROS production was evaluated using the H_2_DCF-DA assay. Data are expressed as mean ± SEM of at least three independent experiments. ****p < 0.0001 vs. vehicle; #*p* < 0.05, ##*p* < 0.01, ####*p* < 0.0001 vs. LPS; One-way ANOVA followed by Tukey post-test for multiple comparisons

Intracellular ROS production was quantified using the H₂DCF-DA fluorescence assay. Exposure of C6 cells to LPS markedly increased ROS levels compared to the control group (Fig. 4). In contrast, treatment with Que 15 µM and 30 µM or Que-3α 15 µM and 30 µM, did not alter baseline ROS production. Furthermore, pretreatment of C6 cells with Que or Que-3α at both tested concentrations significantly reduced LPS-induced ROS production. For Que-3β the lower concentration (15 µM) did not significantly modify ROS levels in LPS-exposed cells compared to the LPS group, however the higher concentration (30 µM) increased ROS production under basal conditions. Additionally, neither concentration of Que-3β was able to reduce LPS-induced ROS levels.

###  Quercetin and Quercetin-3’-α-D-glucoside Prevent Cell Death Induced by LPS

**Fig. 5:**
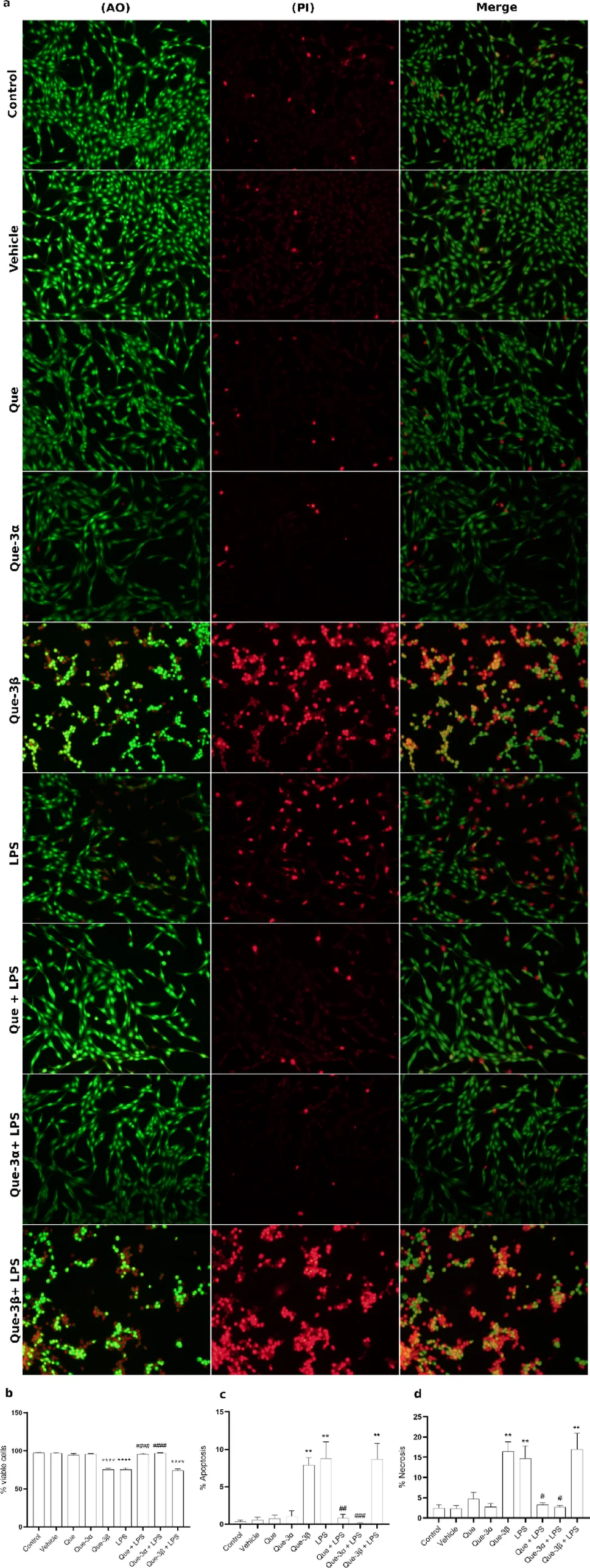
Effect of quercetin (Que), quercetin-3’-α-D-glucoside (Que-3α) and quercetin 3-β-D-glucoside (Que-3β) on cell viability, apoptosis, and necrosis in C6 cells. Cells were pretreated with Que or Que-3α or Que-3β (30 µM) for 2 h, followed by exposure to LPS (10 µg/mL) for 24 h. **A** Representative images (200× magnification), **B** Percentage of viable cells, **C** apoptotic cells, and **D** necrotic cells, determined by acridine orange/propidium iodide (AO/PI) staining. Data are expressed as mean ± SEM (*n* = 3). ***p < 0.001 and ****p < 0.0001 vs. control; ###*p* < 0.001 and ####*p* < 0.0001 vs. LPS. One-way ANOVA followed by Tukey post-test for multiple comparisons

Cell viability, apoptosis, and necrosis were evaluated by AO/PI staining after the treatments (Fig. 5). The results indicate that LPS significantly reduced cell viability compared with the vehicle group (Fig. 5B). In contrast, pretreatment with Que and Que-3α completely prevented LPS-induced cytotoxicity, maintaining cell viability at levels comparable to the control and vehicle group, as observed by the large number of cells with green fluorescence. Treatments with Que and Que-3α alone did not affect cell viability.

The quantification of apoptotic cells showed a significant rise in apoptosis in the LPS-treated group compared to the control group (Fig. 5C), with many cells exhibiting nuclear condensation. Pretreatment with Que and Que-3α significantly reduced the percentage of apoptotic cells compared with LPS, restoring values close to those observed in the control and vehicle groups. Neither Que nor Que-3α alone induced apoptosis.

Similarly, exposure to LPS markedly increased necrotic cell death compared with the control (Fig. 5D), as observed by the large number of cells in red fluorescence. Pretreatment with Que and Que-3α effectively attenuated this effect. No significant differences were observed between the Que and Que-3α groups, indicating comparable protective effects. Representative images are shown in Fig. 5D, where viable cells appear green, apoptotic cells show condensed nuclei, and necrotic cells are stained red.

Unlike Que and Que-3α, treatment with Que-3β alone markedly reduced the percentage of viable cells and significantly increased necrotic cell death compared with the control group. Representative fluorescence images corroborated these findings, showing an increased number of PI-positive cells in the Que-3β-treated group. Upon LPS stimulation, pretreatment with Que-3β failed to protect against LPS-induced cell death. The percentages of apoptotic and necrotic cells remained comparable to those observed in the LPS group, indicating that the β-glycosylated derivative did not prevent the loss of membrane integrity or the increase in apoptotic cell death induced by the inflammatory stimulus.

### Effect of Quercetin and Quercetin-3’-α-D-glucoside on the Expression of Inflammatory Markers Induced by LPS

Since Que-3β did not demonstrate a protective effect in the analyses described above, it’s the effects on the expression of inflammatory markers was conducted only for Que and Que-3α.

**Fig. 6:**
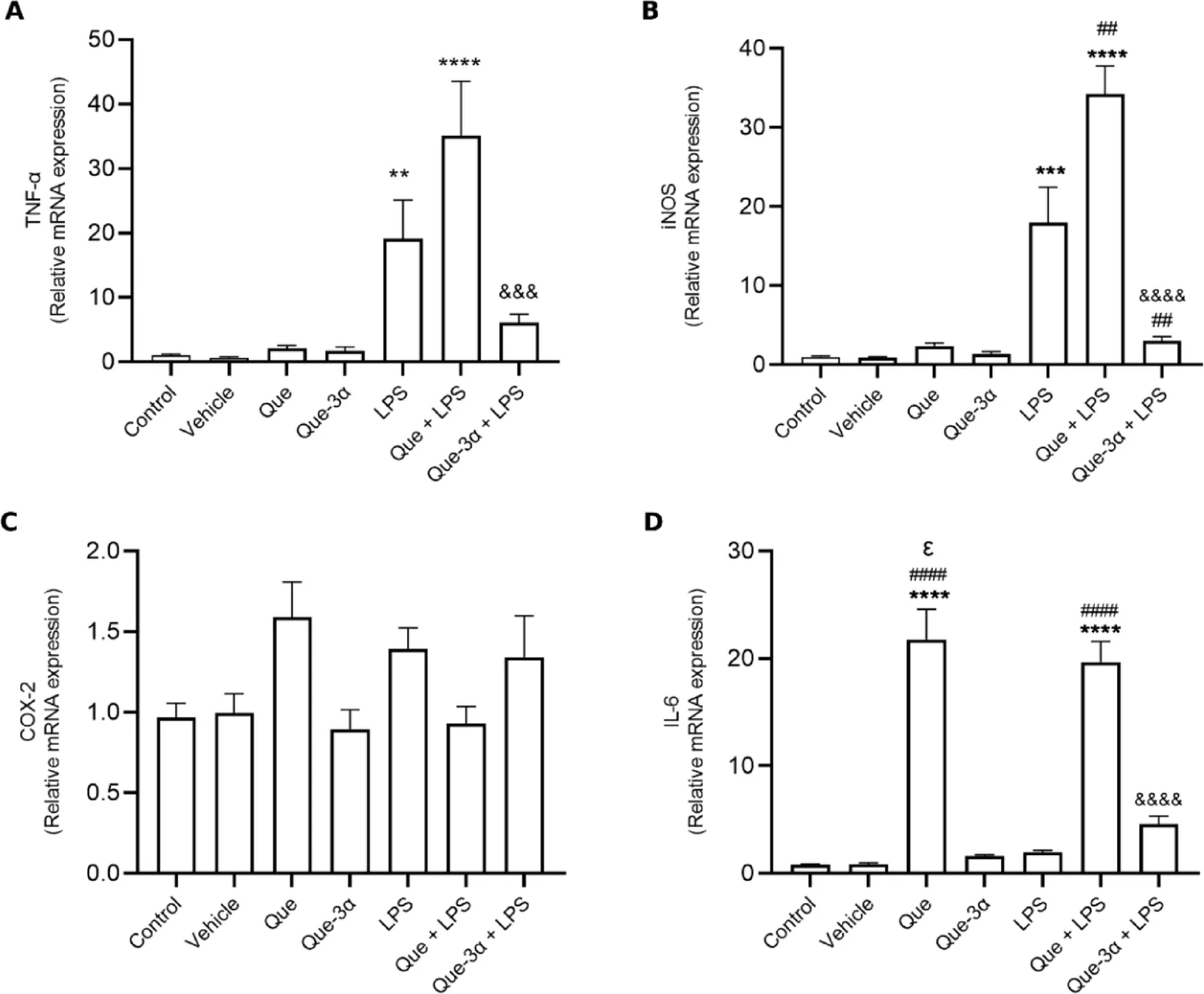
Effects of quercetin (Que) and quercetin-3’-α-D-glucoside (Que-3α) on the mRNA expression of pro-inflammatory mediators in C6 cells. Cells were pretreated for 2 h with Que or Que-3α (30 µM), followed by stimulation with LPS (10 µg/mL) for 24 h. Relative mRNA expression levels of **A** TNF-α, **B** iNOS, **C** COX-2, and **D** IL-6 were determined by quantitative real-time PCR (qPCR), normalized to the mean of β-actin and Tubulin 1, and expressed relative to the control group. Data are presented as mean ± SEM (*n* = 6). **p < 0.01, ***p < 0.001, ****p < 0.0001, vs. vehicle; #*p* < 0.05, ####*p* < 0.0001 vs. LPS; &*p* < 0.05, &&&*p* < 0.001 indicate significant differences between Que + LPS and Que-3α + LPS. ɛ *p* < 0.05 indicate significant differences between Que and Que-3α. One-way ANOVA followed by Tukey post-test for multiple comparisons

LPS exposure significantly increased TNF-α mRNA expression compared to the vehicle group (Fig. 6A). Similarly, the Que + LPS group markedly elevated TNF-α levels compared to the vehicle. In contrast, the combination of Que-3α with LPS did not differ from the vehicle group or from the LPS group, however, TNF-α expression was significantly lower in this group compared to the Que + LPS group. Regarding treatments with the isolated compounds, no significant differences were observed compared to the vehicle.

LPS exposure also significantly increased iNOS mRNA expression compared to the vehicle group (Fig. 6B). This increase was further enhanced in the Que + LPS group, which showed higher levels than both the LPS group and the vehicle. In contrast, the combination of Que-3α with LPS did not differ from the vehicle group and showed significantly reduced expression compared to both the LPS group and the Que + LPS group. No significant differences were observed between the isolated compounds and the vehicle.

Regarding COX-2 mRNA expression, no significant differences were observed among the experimental groups (Fig. 6C). Expression levels remained unchanged following treatment with LPS, Que, or the Que-3α, either alone or in combination.

Que treatment alone significantly increased IL-6 mRNA expression compared to the vehicle group (Fig. 6D). Similarly, Que + LPS resulted in elevated IL-6 levels compared to the vehicle. In contrast, LPS did not alter IL-6 expression compared to the vehicle group. Que-3α, either alone or in combination with LPS, did not differ from the vehicle group. Additionally, IL-6 expression was significantly reduced in the Que-3α + LPS group compared to the Que + LPS group (Fig. 6D).

## Discussion

Research has suggested that glucosylation of flavonoids, such as Que, may improve selected physicochemical and biological properties [48]. In this field, our research group recently synthesized and characterized new α-glucosylated derivatives of Que and evaluated their cytotoxicity in SNC cell lines [40, 41]. Among them, the derivative Que-3α showed the highest yield, as well as interesting pharmacokinetic properties and low cytotoxicity compared to Que. Therefore, in the present study, we investigated the effects of Que and the Que-3α derivative in an LPS-induced inflammation model using C6 astroglioma cells.

Our findings demonstrated that both Que and Que-3α significantly mitigated mitochondrial damage and the increase of ROS induced by LPS. In contrast, Que-3β exerted protection against mitochondrial dysfunction only at the lower concentration, while failing to reduce LPS-induced ROS production. Furthermore, the higher concentration of Que-3β increased basal ROS levels, suggesting that the biological activity of the β-glucosylated derivative differs from that of Que and the novel α-glucosylated derivative.

LPS, an endotoxin from Gram-negative bacteria, is commonly utilized in neuroinflammation experimental models. It primarily activates the inflammatory cascade by binding to Toll-like receptor 4 (TLR4), which activates the NF-κB signaling pathway. This process leads to the production of pro-inflammatory mediators and high ROS production in mitochondria [49]. Frequently, LPS-induced inflammation in experimental models is used to study molecules with anti-inflammatory potential, such as flavonoids [26, 50].

Previous studies have shown that Que can preserve mitochondrial function and reduce oxidative stress in several in vitro inflammation models [51, 52]. In the study by Sun [53] using LPS-activated BV-2 microglial cells, Que pretreatment (12.5 µM) activated the Nrf2/HO-1 pathway and decreased oxidative stress markers, thus safeguarding mitochondrial integrity. In macrophage RAW 264.7 cells and other cell types, concentrations around 10 µM were linked to the restoration of mitochondrial membrane potential, the maintenance of ATP levels, and the activation of the Sirtuin 1 and Peroxisome Proliferator-Activated Receptor Gamma Coactivator 1-alpha (SIRT1/PGC-1α) pathway, previously identified mechanisms that support mitochondrial biogenesis and antioxidant responses [54].

The antioxidant activity of Que has been attributed, at least in part, to the presence and arrangement of hydroxyl groups within its chemical structure, which favor electron donation and radical scavenging [55]. In the present study, both Que and Que-3α significantly reduced LPS-induced intracellular oxidative stress under the experimental conditions employed. However, the molecular mechanisms responsible for this effect were not investigated, and therefore the involvement of antioxidant pathways, such as Nrf2 signaling, cannot be inferred from the present findings.

Interestingly, the biological behavior of Que-3β differed from that observed for Que-3α. As mentioned earlier, although the lower concentration of Que-3β preserved mitochondrial activity following LPS exposure, neither concentration attenuated LPS-induced ROS production, and the higher concentration even increased basal ROS levels. These findings suggest that glucosylation per se is not sufficient to preserve or enhance the antioxidant properties of Que. Although few studies have specifically compared α- and β-glucosides of Que, accumulating evidence suggests that the biological effects of glucosylated derivatives depend on subtle structural characteristics, including the nature, position, and potentially the stereochemical configuration of the glycosidic bond, which may influence molecular conformation and interactions with biological targets [56].

Previous studies have shown that glucosylated derivatives of Que generally exhibit improved physicochemical properties compared with the aglycone, including enhanced water solubility, improved cellular uptake, and prolonged antioxidant activity [57–61]. For example, Liangqin Xie et al. [61] compared aglycone flavonoids (Que and luteolin) with their glucosylated counterparts (isoquercitrin and orientin) during in vitro and in vivo digestion. Although the aglycones displayed greater initial antioxidant activity, their effects declined rapidly, whereas the glucosides exhibited greater stability and sustained antioxidant capacity. These findings support the hypothesis that glucosylation may protect the flavonoid structure against oxidative degradation, thereby contributing to prolonged biological activity.

Our results are consistent with these observations. Que-3α produced a more pronounced reduction in LPS-induced oxidative stress than Que, suggesting that glucosylation may have contributed to the improved suppression of LPS-induced oxidative stress observed under the present experimental conditions. However, our findings also demonstrate that the biological effects of glucosylation depend not only on the presence of the glucose moiety but also on the stereochemistry of the glycosidic linkage. Although Que-3β preserved mitochondrial activity at the lower concentration, it failed to attenuate LPS-induced ROS production and, at the higher concentration, even increased basal ROS levels. In contrast, Que-3α consistently reduced oxidative stress and exhibited a more favorable biological profile.

The distinct activities observed between Que-3α and Que-3β are particularly noteworthy because the two molecules differ only in the stereochemical configuration of the glycosidic bond. Such subtle stereochemical differences are known to influence molecular conformation, hydrogen-bonding patterns, physicochemical properties, and interactions with enzymes and biological targets, thereby affecting the stability, bioavailability, and biological activity of glycosylated flavonoids [62, 63].

Our results indicated that LPS not only induced mitochondrial dysfunction and elevated oxidative stress, but also markedly diminished cell viability, facilitating the emergence of cells exhibiting morphological characteristics of apoptotic death and compromised membrane integrity. These findings suggest the occurrence of distinct patterns of cell death; however, this method does not distinguish between passive necrosis and programmed necrotic forms such as necroptosis, which require confirmation using specific molecular markers.

More detailed studies on the apoptotic effects of LPS in C6 cells have shown that this agent is able to increase the expression of Bax, cytochrome C (CytC), and cleaved caspase-3, which are key components of the intrinsic (or mitochondrial) apoptosis pathway, while simultaneously reducing the levels of the anti-apoptotic protein Bcl-2 [64]. Collectively, these studies suggest that LPS-induced mitochondrial dysfunction is associated with disruption of the electron transport chain, increased ROS generation, mitochondrial permeability transition, cytochrome c release, and activation of apoptotic cascades [65].

In this study, both Que and Que-3α showed antiapoptotic properties. These findings indicate that the neuroprotective properties of Que and its derivatives extend beyond free radical scavenging and may also encompass the maintenance of mitochondrial integrity and the enhancement of cell survival by disrupting the LPS-induced cell death cascade. In contrast, Que-3β failed to confer similar cytoprotection and, at the highest concentration tested (30 µM), further increased apoptosis and necrosis.

Prior in vitro investigations in glial and neuronal cell lines suggest that the anti-apoptotic properties of Que may be associated with its capacity to regulate various intracellular signaling pathways. In neuronal cells, Que has been shown to activates the transcription factor Nrf2 and modulates the Akt and MAPK pathways, resulting in decreased expression of Bax and cleaved caspase-3, and increased levels of the anti-apoptotic protein Bcl-2 [66, 67]. In addition to its anti-apoptotic properties, Que also provides protection against necrotic cell death. An in vitro study using human colon epithelial T84 cells exposed to H_2_O_2_, which induced necrotic cell death, observed that Que prevented necrosis in a concentration-dependent manner (5–20 µM). Additionally, Que may impede other critical cellular processes that result in necrosis, including the activation of poly (ADP-ribose) polymerase (PARP) [68, 69]. Although these mechanisms provide plausible explanations for the protective effects of Que reported in the literature, the present study did not evaluate signaling pathways such as Nrf2, Akt, or MAPK, and therefore no mechanistic conclusions can be drawn regarding their involvement.

In the qPCR analyses, it was possible to observe that the exposure of C6 cells to LPS increased the expression of the pro-inflammatory cytokines such as TNF-α and iNOS. These findings are consistent with the established role of LPS as an activator of the TLR4/MyD88/NF-κB pathway in glial cells [70], leading to the transcription of classical inflammatory genes. The C6 astrocytic cell line functionally expresses the pattern-recognition receptor TLR4 and its adaptor MyD88, leading to activation of the transcription factor NF-κB in response to LPS stimulation [71]. This activation promotes an increase in pro-inflammatory cytokines and other mediators, making this model suitable for investigating modulators of the TLR4/MyD88/NF-κB pathway in the context of neuroinflammation.

Neither Que nor the glucosylated derivative were able to reduce TNF-α expression in this model. This pattern has also been reported in other studies. The work by Lee et al. [72], although conducted in a different cell model, presented similar results. The authors used RAW264.7 macrophages pretreated for 2 h with Que at different concentrations (6.25, 12.5, and 25 µM), followed by LPS treatment (1 µg/mL) for 24 h, and likewise did not observe any effect of the flavonoid on the levels of TNF-α. A similar result was reported by Kim et al. [73], in which Que (10 and 15 µM) inhibited IL-1β, IL-18, and IL-6, but did not modify TNF-α levels in the cellculture treated with LPS (1 µg/mL) for 20 h. Another study that evaluated and compared the effects of Que and the derivative quercetin-3-O-β-D-glucuronide in RAW264.7 macrophages challenged with LPS found a significant reduction in TNF-α only at the concentration of 50 µM of Que, while no effect was observed with the derivative at the tested concentrations (up to 100 µM) [74]. However, the Que-3α + LPS significantly reduced TNF-α levels compared to the Que + LPS group, highlighting its ability to attenuate the potentiating effect induced by Que under inflammatory conditions. This difference can be attributed to the structural modifications introduced by glycosylation, which may reduce the redox reactivity of the molecule [61].

The expression of COX-2 remained unchanged across all experimental groups, a result that was also observed in the study by Lee et al. [72]. The authors discussed that the regulatory mechanism involved in COX-2 activation differs from that of other inflammatory mediators. For example, the distinct responses of the iNOS and COX-2 genes to LPS can be explained by the specific features of their promoters, which determine the signaling pathways required for their activation [75]. Although both iNOS and COX-2 are classically described as LPS-inducible genes, the literature shows that their promoters exhibit different degrees of dependence on transcription factors, which may result in variable responses depending on the cell type and the intensity of the stimulus [75–77].

The iNOS promoter contains several regulatory elements, including NF-κB, AP-1, STAT, and C/EBPβ, making this gene more sensitive to the inflammatory activation triggered by LPS [78], which is consistent with our findings showing that LPS treatment significantly increased its expression. In contrast, the COX-2 promoter, although also dependent on NF-κB, requires the participation of CRE (cAMP Response Elements), a pathway that is not always fully activated by LPS in astrocytic-like cells such as C6 [78, 79]. Thus, while LPS efficiently activates the pathways required to induce iNOS and other pro-inflammatory cytokines, it may not activate all transcription factors essential for COX-2 expression.

Increased iNOS expression was observed after LPS exposure, as expected given its well-established role as a potent inducer of inflammatory responses. Although Que is widely described as an anti-inflammatory and antioxidant compound, it can also exhibit pro-oxidant properties under specific conditions. In the present study, pretreatment with Que in combination with LPS unexpectedly potentiated iNOS expression compared to LPS alone, suggesting a pro-inflammatory effect of Que in this context. In contrast, the Que-3α derivative effectively reduced iNOS expression, suggesting that α-glycosylation may influence the inflammatory response, although the molecular basis for this effect remains to be established. Reduced iNOS levels have also been reported with other Que derivatives, such as quercetin-3-O-β-D-glucuronide, which, after 1 h of pretreatment in RAW264.7 macrophages stimulated with LPS, reduced both enzyme expression and NO levels [74].

The 24-hour LPS exposure did not increase IL-6 levels, it is possible that a longer exposure would have resulted in a more pronounced effect. Several in vitro studies indicate that, after prolonged LPS stimulation for more than 24 h, IL-6 secretion and gene expression rise substantially, supporting the idea that IL-6 functions as a late-phase mediator in the inflammatory response [80]. Several studies demonstrate that IL-6 upregulation is predominantly a late inflammatory response. Macrophages and monocyte-derived cell lines increase IL-6 production only after 48 h of LPS exposure [81]. In mesenchymal stem cells and hiPSC-derived cardiomyocytes, IL-6 levels persist or intensify at 48 h, reflecting feedback and autocrine pathways such as JAK/STAT [82, 83]. Bone-marrow-derived macrophages similarly maintain elevated IL-6 and other mediators after 48 h of stimulation [84]. Consistent findings across multiple cell types reinforce that prolonged LPS exposure is suitable for investigating mechanisms characteristic of the late phase of inflammation [85].

IL-6 can act as both a pro-inflammatory and an anti-inflammatory cytokine, depending on the signaling pathway activated, the specific cell type, and the physiological or pathological context [86]. Although IL-6 is typically associated with pro-inflammatory responses, it also contributes to neuronal differentiation and synaptic plasticity and may activate survival-related pathways after injury. However, dysregulation of IL-6 has been implicated in cognitive impairments, neuronal degeneration, and cell death in various neurological disorders [87].

Several mechanisms reported in previous studies could potentially explain the increase in IL-6 observed after Que treatment. First, Que may have activated signaling pathways associated with IL-6 induction, particularly MAPK (ERK/p38) and JAK/STAT (STAT3). This pattern is consistent with the hormetic behavior of flavonoids [88], which can activate MAPKs at moderate doses or under mild cellular stress. Second, Que is known to interfere with the activity of tyrosine-kinase receptors such as EGFR, which in turn modulates the intracellular kinases mentioned above (ERK1/2 and p38 MAPK). Activation of these pathways is recognized as a driver of IL-6 transcription in astrocytes and glial cell lines, independent of classical NF-κB activation [89]. However, because these pathways were not directly evaluated in the present study, their contribution to the increased IL-6 expression observed here remains speculative and requires further experimental investigation. Overall, the present findings are consistent with previous reports describing the antioxidant, anti-apoptotic, and cytoprotective properties of Que and suggest that its α-glucosylated derivative, Que-3α, may offer advantages in specific biological responses. Notably, under the experimental conditions employed, Que-3α exhibited greater modulation of iNOS expression under the experimental conditions employed. This difference suggests that α-glycosylation may influence the biological behavior of Que. Although previous studies have reported that glucosylation can improve physicochemical properties such as stability, solubility, and intracellular availability, which have previously been identified as key factors contributing to the enhanced biological performance of modified flavonoids [48, 90], these parameters were not evaluated in the present study. However, it is important to highlight that these findings support the idea that glucosylation can strengthen mechanisms already attributed to Que. Although inflammatory mediators were not quantified at the protein level, the consistent effects observed across cell viability, ROS production, and inflammatory gene expression analyses provide complementary evidence supporting the anti-inflammatory activity of both compounds. Therefore, the convergence of these independent endpoints strengthens the overall interpretation of the findings. Nevertheless, future studies incorporating protein-based approaches may further clarify the relationship between transcriptional regulation and downstream inflammatory responses.

## Conclusion

The data obtained in this study reinforce the known biological properties of Que but confer particular relevance to the α-glucosylated derivative synthesized by our group, given that reports of α-configured glucosides for this flavonoid are rare in the literature and, to date, no evaluation of their biological activity in neuroinflammation models has been conducted. Although β-glycosylated derivatives are common in nature, the α configuration represents a less explored structural modification, and its pharmacological implications remain unknown. The results provide the first evidence of how this structural modification influences selected cellular responses associated with inflammation under the experimental conditions employed.

These findings suggest that α-glucosylation preserves the cytoprotective and antioxidant effects of Que under the present experimental conditions and may influence specific responses. The inclusion of the β-glucosylated derivative further demonstrated that the biological effects of Que derivatives depend not only on glucosylation itself but also on the stereochemical configuration of the glycosidic linkage, highlighting the importance of structure–activity relationships in the rational design of new flavonoid derivatives. Given the pharmacokinetic limitations of Que, which remain a major barrier to its therapeutic application, the data presented here provide evidence that α-glucosylation is a promising structural strategy for generating derivatives with improved bioactive potential and, consequently, future applicability in neuroinflammation models.

It is important to note that, although the *in vitro* findings are promising, additional studies are required to confirm the therapeutic potential of Que-3α. To determine whether the effects observed in C6 cells are reproduced in more complex biological systems, future investigations should include *in vivo* models of neuroinflammation and neurodegeneration. This study demonstrated significant cytoprotective effects, reduced intracellular oxidative stress, and modulation of selected inflammatory markers. Under the experimental conditions employed, the anti-inflammatory advantage of Que-3α over Que was observed primarily in the modulation of iNOS mRNA expression, whereas no consistent differences were detected for the other pro-inflammatory markers evaluated. Furthermore, despite the beneficial effects of Que and its α-glucosylated derivative, the precise molecular mechanisms underlying these actions remain to be fully elucidated.

Future investigations should focus on the evaluation of key inflammatory signaling pathways, including NF-κB, MAPK (p38/JNK), and PI3K/STAT, as well as upstream regulators associated with LPS/TLR4 signaling and antioxidant signaling networks such as the Nrf2/Keap1/HO-1/NQO1 axis. Furthermore, given the well-established crosstalk between oxidative stress and inflammatory responses, the potential involvement of sirtuin-related pathways, particularly SIRT1-mediated regulation of NF-κB activity and cellular redox homeostasis, also warrants investigation. In addition, mechanistic studies are essential to identify the molecular targets and signaling networks involved in the actions of the α-glucosylated derivative, particularly regarding its interactions with inflammatory mediators, redox-regulating systems, and glial cell responses. Such analyses will be critical for establishing the translational relevance of this compound and for determining whether α-glucosylation represents a broader strategy for enhancing the therapeutic potential of flavonoids for the prevention and treatment of neuroinflammatory disorders.

## Data Availability

No datasets were generated or analysed during the current study.
